# Bryophytes and Organic layers Control Uptake of Airborne Nitrogen in Low-N Environments

**DOI:** 10.3389/fpls.2017.02080

**Published:** 2017-12-04

**Authors:** Alexandra Bähring, Andreas Fichtner, Uta Friedrich, Goddert von Oheimb, Werner Härdtle

**Affiliations:** ^1^Institute of Ecology, Leuphana University of Lüneburg, Lüneburg, Germany; ^2^Institute of General Ecology and Environmental Protection, Technische Universität Dresden, Tharandt, Germany

**Keywords:** *Calluna vulgaris*, critical load, heathland, nitrogen cycling, nitrogen retention, nitrogen saturation

## Abstract

The effects of atmospheric nitrogen (N) deposition on ecosystem functioning largely depend on the retention of N in different ecosystem compartments, but accumulation and partitioning processes have rarely been quantified in long-term field experiments. In the present study we analysed for the first time decadal-scale flows and allocation patterns of N in a heathland ecosystem that has been subject to airborne N inputs over decades. Using a long-term ^15^N tracer experiment, we quantified N retention and flows to and between ecosystem compartments (above-ground/below-ground vascular biomass, moss layer, soil horizons, leachate). After 9 years, about 60% of the added ^15^N-tracer remained in the N cycle of the ecosystem. The moss layer proved to be a crucial link between incoming N and its allocation to different ecosystem compartments (in terms of a short-term capture, but long-term release function). However, about 50% of the ^15^N captured and released by the moss layer was not compensated for by a corresponding increase in recovery rates in any other compartment, probably due to denitrification losses from the moss layer in the case of water saturation after rain events. The O-horizon proved to be the most important long-term sink for added ^15^N, as reflected by an increase in recovery rates from 18 to 40% within 8 years. Less than 2.1% of ^15^N were recovered in the podzol-B-horizon, suggesting that only negligible amounts of N were withdrawn from the N cycle of the ecosystem. Moreover, ^15^N recovery was low in the dwarf shrub above-ground biomass (<3.9% after 9 years) and in the leachate (about 0.03% within 1 year), indicating still conservative N cycles of the ecosystem, even after decades of N inputs beyond critical load thresholds. The continuous accumulation of reactive forms of airborne N suggests that critical load-estimates need to account for cumulative effects of N additions into ecosystems.

## Introduction

Atmospheric inputs of reactive nitrogen (comprising all N species with the exeption of N_2_; Galloway and Cowling, 2002) have tripled since the nineteenth century, resulting in unprecedented impacts on the N status of ecosystems (Galloway and Cowling, 2002; De Schrijver et al., 2011). Since N is the most limiting nutrient in many natural and semi-natural environments, high N loads may affect important ecosystem functions such as biomass production and the complex interplays within and between plant species, but also their susceptibility to abiotic and biotic stressors such as drought, frost events and herbivory (Bobbink et al., 2010). As a consequence, atmospheric N deposition constitutes one of the most substantial threats to biodiversity today (Sala et al., 2000; Phoenix et al., 2012). This pertains to N-limited ecosystems with conservative N cycles in particular, because many species that are typical of these environments are physiologically adapted to low N availability, for example due to high N use efficiency or mycorrhizal associations (Aerts, 1999; Bobbink et al., 2002; Phoenix et al., 2012).

European heathland ecosystems are typically low-N environments (Härdtle et al., 2006). These heathlands host a huge proportion of the plant and animal diversity typical of open acidic sites, but also provide a wide range of ecosystem services (e.g., groundwater recharge, C-storage, recreation; De Graaf et al., 2009; Fagúndez and Izco, 2016). Agricultural intensification and elevated atmospheric N inputs, however, have contributed to a distinct decline in European heathland area over recent decades (Rose et al., 2000; Fagúndez, 2012; Southon et al., 2012), and nowadays remaining areas are protected by several international protection acts (such as the Natura2000 Habitat Council Directive 92/43/EEC) (Rose et al., 2000; Vandvik et al., 2005; Southon et al., 2012).

Many lowland heaths of NW Europe are characterised by the dominance of the dwarf shrub *Calluna vulgaris* (L.) Hull (henceforth referred to as *Calluna*). This species is competitively superior to grasses (e.g., *Molinia caerulea, Deschampsia flexuosa*) under low-N conditions, but becomes less competitive with increasing N availability (Alonso et al., 2001; Friedrich et al., 2011a; Fagúndez, 2012). At N-fertilised sites, a succession towards grass dominance is observable when the dwarf shrub canopy opens as a result of overaging, heather beetle outbreaks or frost and drought damage (Berdowski and Zeilinga, 1987; Bobbink et al., 2010). This process accelerates under increasing N availability, attributable to faster rates of N cycling and plant growth, and finally, shortened life-cycles of *Calluna* (Berendse, 1990; Power et al., 1998; Carroll et al., 1999). Surprisingly, dwarf shrub growth still is N-limited in some heathlands, despite decadal N inputs above critical loads (von Oheimb et al., 2010; Friedrich et al., 2011b). Such findings could be related to both time- and space-dependent allocation and storage processes of airborne N loads, or to underestimated N losses from the N pool of the ecosystem. It is, for example, conceivable that the spodic horizon (B-horizon) of podzols can accumulate considerable amounts of N in organic compounds (fulvic and humic acids; Nielsen et al., 2000; Friedrich et al., 2011b), all of which are to a large extent insoluble and not readily bio-available (Hagedorn et al., 2005). In this way, N inputs might be (at least partly) withdrawn from the N cycle and thus become biologically inactive. However, we still have limited evidence for the long-term efficacy of atmospheric N loads on the functioning of heathlands as well as other ecosystems. In addition, decadal-scale patterns of N flows and sequestration have rarely been quantified, restricting a mechanistic understanding of N-fertilisation effects on ecosystem functions. This in turn emphasises the value of long-term studies that trace the allocation of N to or between ecosystem compartments on a decadal scale (Silvertown et al., 2010).

Many N-budget calculations from long-term fertilisation experiments have provided valuable knowledge about shifts in N stores of different ecosystem compartments (Power et al., 1998; Pilkington et al., 2005; von Oheimb et al., 2010), but were unable to quantify the origin of N gains and losses over time and, thus, N flows between ecosystem compartments such as plant biomass, organic layers or mineral soil horizons. ^15^N tracer studies have the potential to overcome this problem and allow for a temporal quantification of N flows (Kleinebecker et al., 2009), as has been demonstrated for grassland and forest ecosystems (Gerzabek et al., 2004; Nadelhoffer et al., 2004). Quantitative analyses of the fate of airborne N loads including different ecosystem compartments are important for an understanding of ecosystem responses to long-term N inputs at the individual plant and community level, but may also provide insights into mechanisms underlying shifts in species composition and losses (Friedrich et al., 2011b). Such information can further help to develop adaptive management strategies for semi-natural N-limited ecosystems and the species they host, particularly in the face of ongoing N inputs and their contribution to biodiversity loss (Maskell et al., 2010).

The objective of the present study was to quantify decadal-scale patterns of N accumulation and partitioning in an N-limited environment that has been subject to high airborne N inputs over decades. We analysed N flows (in terms of ^15^N tracer enrichment and recovery) to and between different ecosystem compartments, using an experiment that was established in a heathland ecosystem in 2007 (Friedrich et al., 2011b) and continued until 2015. We were particularly interested in the impact of the moss layer on the N cycle of the ecosystem, because this compartment is considered an important sink for incoming N (Proctor, 2008), but also might release N to other compartments, for example via downward transports in the soil profile. In this way, the moss layer could contribute to N enrichment in deeper soil horizons (Aldous, 2002; Proctor, 2008; Startsev et al., 2008). Moreover, we asked whether there are indications of N-saturation, or whether ecosystem-internal sinks for N or unknown pathways of N losses might counteract an accumulation of airborne N inputs. We hypothesised that (i) the moss layer constitutes a crucial component of the N cycle of the ecosystem, based on its capture-release-function for airborne N, (ii) the podzol-B-horizon serves as an important long-term sink for N, and (iii) the heathlands studied still have a high capacity to sequester pollutant N.

## Materials and methods

### Study site and experimental design

Our study site is located in the Lüneburg Heath nature reserve in NW Germany (Lower Saxony, Schneverdingen, 53°15′N, 9°58É, 105 m a.s.l.), the site of the largest complex of heathlands (about 5,000 ha) in NW Germany. The study sites is characterised by Pleistocene sandy deposits with a weak relief and nutrient-poor podzol soils. Topsoil-pH_H2O_ values range between 3.3 and 3.5 (Härdtle et al., 2006). The climate is of a humid sub-oceanic type (mean precipitation: 811 mm year^−1^, annual mean temperature: 8.4°C; Niemeyer et al., 2005)., Heathlands of the nature reserve are dominated by the dwarf shrub *Calluna* (cover >80%). The soil surface is covered by mosses (mean cover 93%), with *Hypnum judlandicum* and *Pleurozium schreberi* being the prevailing species. The current background atmospheric N deposition in the study area amounts to 23 kg ha^−1^ year^−1^ (von Oheimb et al., 2010 for more detailed information on the quantity of different N-forms deposited in NW Germany and in the study area see Gauger et al. (2000) and Niemeyer et al. (2005), respectively).

The present study made use of the experimental setup of Friedrich et al. (2011b) established in 2007. The experiment comprises a total of seven randomly selected plots, each of which was divided into two subplots (2 × 4 m in size). At the beginning of the experiment (June 2007), in one subplot (per plot) a ^15^N tracer was added in liquid form (pulse labeling with 100 mg ^15^${\text{NH}}_{4}^{15}$NO_3_ (98 at.%) using a special spray bottle that allowed for a fine nebulisation of the liquid applied), and the corresponding subplot was used to determine the natural ^15^N abundance (these subplots are henceforth referred to as labelled and non-labelled subplots, respectively). Labelled and non-labelled subplots were separated by a buffer zone 1 m in width to avoid cross contamination after ^15^N tracer addition (for a detailed description of the ^15^N application procedure see Friedrich et al., 2011b).

All analyses performed during the present study refer to a time span of 7 years, with all samples collected between 2009 and 2015 (with the exception of one plot, which was sampled from 2008 to 2015, as well as litter and leachate samples; see description below). To present a complete overview of N flows and partitioning since the setup of the experiment in 2007, we included analyses of Friedrich et al. (2011b) in the present study (i.e., analyses from 2007 to 2008). In this way, we were able to document compartment-related shifts in ^15^N abundance, enrichment and recovery for a 9-year timespan (2007–2015).

### N allocation patterns in biomass and soil

To quantify N allocation patterns in the biomass and soil we sampled the following ecosystem compartments annually: above-ground biomass of *Calluna* (current year and 1- to 2-year-old shoots), moss layer, and the soil O-, A- and B-horizons (with the latter two representing the albic and spodic horizons, respectively, both of which are typical of podzol soils; nomenclature according to the IUSS Working Group WRB, 2015) in labelled and non-labelled subplots in each August from 2008 to 2015, corresponding to the flowering period of *Calluna*. Twenty randomly chosen current year shoots as well as 1- to 2-year-old shoots of *Calluna* from the top of randomly chosen *Calluna* plants were cut with scissors and were pooled to one sample per subplot. Six randomly chosen squares of the moss layer (2 × 2 cm in size) were sampled and bulked to one sample per subplot. Organic soil samples (O-horizon; mean thickness: 2.7 cm) were cut with knives (six squares of 2 × 2 cm of the entire horizon depth per subplot) and separated from fresh fallen litter. Mineral soil samples were taken from the A- and B-horizon with a soil auger (Pürckhauer, EcoTech, Bonn, Germany) from six randomly chosen spots per subplot and bulked to one sample each mean thickness of A- and B-horizons: 7.1 cm and 7.5 cm, respectively). The annual litter production of *Calluna* was quantified in labelled and non-labelled subplots by means of fine-mesh nets stretched under one randomly selected plant per subplot and sampled during winter between the end of February and the beginning of April (depending on the duration of snow cover) from 2008 to 2016.

To obtain an overview of the quantities of ^15^N accumulated in older *Calluna* twigs and stem wood (age >2 years) and *Calluna* below-ground biomass (i.e., roots), but to avoid an interference of plant growth in plots, these compartments were sampled only twice, in August 2014 and 2015. To this end, six randomly chosen stems of *Calluna* per subplot were cut with clippers at ground level (excluding current year and 1- to 2-year-old shoots). For root analysis, we sampled the O-horizon at six randomly selected sites per subplot (3 × 3 cm samples from across the entire organic layer).

Soil and root samples were stored in a freezer (−18°C) until analysis. Prior to analysis, above-ground biomass and soil samples were air-dried, and the latter one sorted and sieved (2 mm). Root samples were washed using a 0.2 mm sieve to remove soil particles and then dried (48 h at 80°C). All samples were ground with a mixer mill (MM 400, Retsch, Haan, Germany) and re-dried at 105°C for weighing. Total N contents and δ^15^N were determined with a continuous flow elemental analyser-isotopic ratio mass spectrometer (vario EL cube, Elementar, Hanau, Germany, coupled to an Isoprime IRMS, Isoprime Ltd., Cheadle Hulme, UK; note that total N analyses of soils hence included microbial biomass N).

### N losses via leaching

N losses via leaching were quantified by means of four pairs of lysimeters, one of which was labelled with 100 mg ^15^${\text{NH}}_{4}^{15}$NO_3_ m^−2^ in 2007 (analogous to the labelling of subplots), and one which received a corresponding amount of deionised water (non-labelled lysimeter; for a detailed description of the setup of lysimeters see Friedrich et al., 2011b). Since the collection and analysis of leachate was very laborious and ^15^N leaching losses (in terms of inorganic N and DON) proved to be negligible at the beginning of the experiment, leaching losses of ^15^N were only quantified on 20 occasions in the period from June 2014 to May 2015 (weekly in June 2014, once every 2 weeks from July to August 2014 and monthly from September 2014 to June 2015). The leachate which accumulated between the sampling occasions was extracted with a temporarily connected vacuum pump. The quantity of leachate per lysimeter was recorded and a subsample of 500 ml was filtered and stored in a freezer (−18°C) for analysis. Samples were carefully defrosted and analysed with a UV/Vis scanning spectrophotometer (UV-3100PC, VWR, Radnor, PA, USA) using the method of Hagedorn and Schleppi (2000). In order to determine δ^15^N values, samples were prepared following the diffusion method of Sebilo et al. (2004) modified according to Friedrich et al. (2011b).

### Calculation of N pools and leaching losses

To calculate ^15^N tracer recovery, the mass of N pools in each ecosystem compartment must be known. Therefore, the total N content of a compartment in labelled subplots was multiplied by its mean N pool mass. Pool masses for the moss and soil compartments were taken from Friedrich et al. (2011b). *Calluna* plants were 10–12 years old at the beginning of the experiment (i.e., early mature phase of life-history; Gimingham, 1972). The continuous increment of *Calluna* above-ground biomass (i.e., total above-ground biomass and total biomass of current year and 1- to 2-year-old shoots) was calculated on the basis of age-dependent growth rates of *Calluna* in the nature reserve, derived from our own measurements (based on biomass harvests at six randomly chosen sites adjacent to the experimental plots and comparisons with data from Friedrich et al., 2011b) in the Lüneburg Heath and complemented by literature data (Gimingham, 1972; Milne et al., 2002). *Calluna* below-ground biomass was also quantified in mature stands adjacent to our plots (at six randomly selected sites, 15 soil samples per site taken with a soil auger 8 cm in diameter and to a soil depth of 15 cm; following the procedure for root analyses described above).

N leaching losses were calculated from the total amount of leachate multiplied by the total N contents (N_inorg_ and DON) of the leachate from labelled lysimeters (Friedrich et al., 2011b).

### Calculation of ^15^N abundance, ^15^N enrichment and ^15^N tracer recovery

^15^N abundance, ^15^N enrichment and ^15^N tracer recovery were calculated according to Fry (2006). ^15^N abundance and the natural ^15^N abundance in labelled and non-labelled subplots, respectively, are represented in the δ notation:

(1) $$\delta^{15}\text{N }[‰]=(\frac{{\text{R}}_{\text{sample}}}{{\text{R}}_{\text{standard}}}-1)\;\cdot \;1,000,$$

where R_sample_ and R_standard_ are the ^15^N/^14^N ratio of the sample and the N_2_ of the atmosphere, which is used as standard, respectively.

^15^N enrichment [‰] represents the isotopic enrichment of a sample from a labelled subplot (δ^15^N_sample_) compared to a sample in a non-labelled subplot (δ^15^N_ref_):

(2) N15enrichment[‰]=δ15Nsample − δ15Nrefδ15Nref + 1,000 · 1,000.

^15^N recovery [g N m^−2^] is the mass of ^15^N tracer recovered in the different ecosystem compartments (biomass, soil horizons, and leachate) in labelled subplots and was calculated as follows:

(3) N15recovery=mpool·at.%15Npool− at.%15Nrefat.%15Ntracer − at.%15Nref,

where m_pool_ is the amount of N in a respective compartment [g N m^−2^], at.%^15^N_pool_ and at.%^15^N_ref_ are the at.%^15^N in the N pool of the samples (or in the leachate) of labelled and non-labelled subplots, respectively, and at.%^15^N_tracer_ is the at.%^15^N of the added ^15^N tracer (Nadelhoffer et al., 2004).

### Statistical analysis

Differences between ^15^N abundances of labelled and ^15^N natural abundances from non-labelled subplots were tested by means of pairwise comparisons of δ^15^N values of each compartment and for each sampling occasion using a Student's *t*-test (α = 0.05). We applied generalised additive mixed models (GAMM) with a log link function and gamma distribution to assess the effects of ecosystem compartments and time (2007–2015) on ^15^N enrichment. Ecosystem compartments were stratified into five main compartments: above-ground biomass of *Calluna* (including current year shoots, 1- to 2-year-old shoots, and current year litter), moss layer, and O-, A- and B-horizons of the soil. To address the skewed response of the ^15^N enrichment data, a gamma probability distribution was preferred, because it retains the structure of the data while accounting for a heteroscedastic error structure (Zuur et al., 2014). We added a value of 0.001 to the ^15^N enrichment data to ensure convergence of the fitting algorithm. The non-linear time effect was modelled using a cubic regression spline with four degrees of freedom to allow some complexity in the ^15^N enrichment function, while avoiding over-fitting the data (Wood, 2006). We used plot as random factor to account for between-plot variability, and a first-order autocorrelation structure (AR-1) nested within plot to account for temporal correlation (Zuur et al., 2009). The resulting GAMM is:

(4) $$EN_{ij\;}=\exp(\alpha +f\left(Time_{ij}\right)+\beta \;Compartment_{ij}+\;b_{i})$$

where *EN*_*ij*_ is the ^15^N enrichment of sample *j* in plot *i* and *EN*_*ij*_ is assumed to follow a Gamma distribution with a log link. α is the intercept, *f* is a smoothing function (cubic regression spline) of the time effect and β is a parametric coefficient of the compartment effect. *b*_*i*_ is a normally distributed random intercept for the plot effect accounting for any dependency between samples from the same plot. The residuals at time *s* are modelled as a function of the residuals of time *s*−1 along with noise (as ε_*s*_ = ρ* ε*_*s*−1_ + η_*s*_, in which ρ is the temporal correlation parameter to be estimated; Zuur et al., 2009). The importance of variables for ^15^N enrichments was assessed by fitting various candidate models accounting for the main effects and their interaction (as *f*(*Time*_*ij*_) *x Comaprtment*_*ij*_) using maximum likelihood estimation (ML). Model selection was based on the Akaike Information Criterion (AIC) and the model with the smallest AIC and highest Akaike weights (*w*_*i*_) was identified as the minimum-adequate model (Burnham and Anderson, 2002). Parameter estimates of the minimum-adequate model were based on the restricted maximum likelihood (REML) method (Zuur et al., 2009). Model assumptions were visually assessed following Zuur et al. (2010). We found no trends in the residuals. To avoid biased parameter estimates, we omitted extreme values (outliers, <2% of the data) for the analyses. Outliers were visually identified by Cleveland dotplots (Zuur et al., 2010). All analyses were conducted in R 3.3.1 (http://www.R-project.org) with the packages nlme (Pinheiro et al., 2016), mgcv (Wood, 2006), and MuMIn (Bartón, 2016).

## Results

### ^15^N abundances and ^15^N enrichment in biomass and soil

δ^15^N values (natural abundance) varied between −8.0 and +8.7‰ across compartments and were lowest in *Calluna* biomass (range between −8.0 and −5.3‰). The moss layer showed δ^15^N values between −6.8 and −5.9‰. In the soils, δ^15^N values increased continuously with soil depth (organic layer: between −4.9 and −3.5‰, A-horizon: between 3.5 and 5.3‰ and B-horizon: between 6.1 and 8.7‰; Table 1).

**Table 1:** ^15^N abundance (δ^15^N in ‰) in non-labelled (Ref.) and labelled subplots (^15^N) of the ecosystem compartments analysed (means, 1 SE (standard error of the mean) in brackets, *n* = 7 samples) during the study period 2007–2015.

**Year**	**Current year shoots**		**1- to 2-year-old shoots**		***Calluna*** **older than 2 years**		***Calluna*** **roots**		**Current year litter**		**Moss layer**		**O-horizon**		**A-horizon**		**B-horizon**	
	**Ref**.	**^15^N**	**Ref**.	**^15^N**	**Ref**.	**^15^N**	**Ref**.	**^15^N**	**Ref**.	**^15^N**	**Ref**.	**^15^N**	**Ref**.	**^15^N**	**Ref**.	**^15^N**	**Ref**.	**^15^N**
2007	−5.67	**129.30**	−5.92	**110.42**					−6.24	54.66	−6.19	**735.74**	−4.53	**19.74**	5.33	**8.16**	8.71	8.74
	(0.51)	(25.56)	(0.47)	(23.28)					(0.16)	(25.18)	(0.22)	(106.17)	(0.35)	(4.22)	(0.40)	(0.48)	(0.43)	(0.69)
2008	−6.54	**65.00**	−7.67	**54.54**					−5.40	**37.55**	−5.93	**535.65**	−4.79	**28.60**	4.26	**9.43**	7.16	8.90
	(0.69)	(10.43)	(0.63)	(11.40)					(0.80)	(9.70)	(0.38)	(55.71)	(0.44)	(6.03)	(1.11)	(0.81)	(1.32)	(0.76)
2009	−6.90	**47.15**	−7.68	**41.55**					−7.66	**40.14**	−6.60	**432.63**	−4.85	**30.62**	4.49	8.50	6.73	8.30
	(0.52)	(5.53)	(0.57)	(6.46)					(0.61)	(11.39)	(0.31)	(63.96)	(0.48)	(4.92)	(1.23)	(0.95)	(1.36)	(1.00)
2010	−6.85	**55.23**	−7.96	**45.77**					−8.02	**38.11**	−6.11	**338.70**	−3.99	**18.57**	4.76	9.26	7.68	9.82
	(0.56)	(7.56)	(0.51)	(6.13)					(0.48)	(8.71)	(0.34)	(32.94)	(0.59)	(2.91)	(1.02)	(1.39)	(1.11)	(1.03)
2011	−6.02	**51.47**	−7.50	**47.30**					−7.60	**36.68**	−6.32	**177.92**	−4.50	**25.12**	4.69	**9.26**	7.21	8.78
	(0.46)	(5.23)	(0.42)	(5.14)					(0.47)	(7.32)	(0.49)	(18.88)	(0.42)	(2.17)	(1.12)	(0.64)	(1.25)	(1.01)
2012	−6.30	**66.69**	−7.07	**59.44**					−6.64	**31.11**	−6.79	**141.69**	−3.52	**38.77**	4.14	**10.55**	6.62	10.24
	(0.51)	(8.60)	(0.46)	(7.55)					(0.64)	(6.29)	(0.18)	(13.06)	(0.53)	(7.28)	(1.18)	(0.25)	(1.28)	(0.46)
2013	−6.55	**49.12**	−7.58	**40.61**					−6.62	**29.57**	−6.75	**109.67**	−3.58	**29.41**	3.91	**13.97**	7.14	10.22
	(0.50)	(6.05)	(0.44)	(5.05)					(0.52)	(5.95)	(0.35)	(9.92)	(0.63)	(2.84)	(1.14)	(2.08)	(1.10)	(0.30)
2014	−6.06	**36.13**	−6.72	**31.92**	−7.00	**38.44**	−5.34	**60.77**	−6.78	**24.09**	−6.74	**50.42**	−3.82	**46.60**	3.53	**15.63**	6.52	10.35
	(0.56)	(5.51)	(0.50)	(4.72)	(0.46)	(4.85)	(0.61)	(10.48)	(0.43)	(5.36)	(0.21)	(7.22)	(0.53)	(7.72)	(1.01)	(3.97)	(1.26)	(0.90)
2015	−6.30	**33.70**	−7.28	**31.90**	−7.40	**34.10**	−5.39	**61.74**	−8.60	**18.33**	−6.63	**40.02**	−3.78	**59.32**	5.17	**10.38**	6.13	9.48
	(0.53)	(6.35)	(0.41)	(5.41)	(0.47)	(5.90)	(0.52)	(5.87)	(0.39)	(4.69	(0.24)	(6.87)	(0.55)	(6.17)	(1.43)	(0.54)	(1.08)	(0.47)

*Significantly (P < 0.05) enhanced δ^15^N values in labelled subplots are indicated in bold*.

As was to be expected, most compartments showed a significant (*P* < 0.05) increase in δ^15^N values (enrichment) after tracer addition (exceptions were the B-horizon, some measurements of the A-horizon and the litter samples in the first year of the experiment). The highest enrichment values were found for the moss layer and *Calluna* shoots (Table 1).

**Figure 1:**
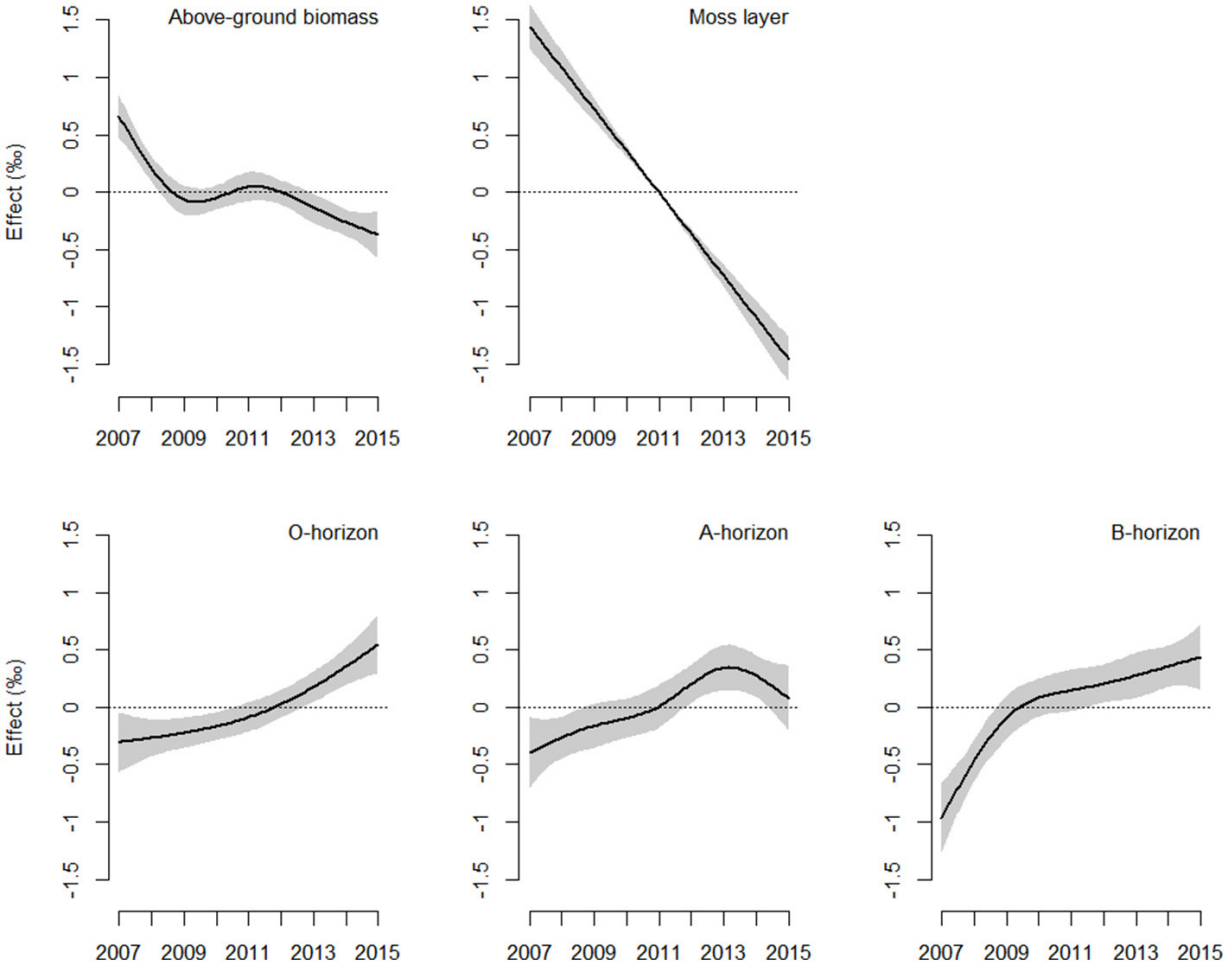
Estimated smoother for time-related shifts in ^15^N enrichment in the ecosystem compartments analysed. The solid line corresponds to fitted relationships of generalised additive mixed models, with shaded areas showing the 95% confidence interval range. The y-axis indicates the contribution of the smoother (effect, ‰) to the fitted values of ^15^N enrichment (see Equation 4 and Table 3; *n* = 7 per compartment and year).

The direction and magnitude of temporal changes in ^15^N enrichment distinctly varied among compartments (Figure 1), as indicated by the strong support for interacting effects of time and compartment (Akaike model weight of 1.0; Table 2). 86 % of the variation in ^15^N enrichment was explained by the interaction between time and compartment, whereas compartment or time effects explained 45 and 7% of the variance, respectively (Table 2). The partial effects of the above-ground biomass of *Calluna* and the moss layer on ^15^N enrichment showed a significant decrease over time (*P* < 0.001 for both compartments; Table 3; Figure 1), while a significant increase of ^15^N enrichment was observed in the soil (O- and B-horizon: *P* < 0.001; A-horizon: *P* < 0.01; Table 3; Figure 1). ^15^N enrichment in the A-horizon also increased within the course of the experiment, but decreased in 2015 (Table 1).

**Table 2:** Model selection statistics for four candidate models describing ^15^N enrichment as a function of ecosystem compartment (above-ground biomass, moss layer, O-, A-, and B-horizons) and time (2007–2015).

**Model**	**Fixed effects**	**ΔAIC**	***w_*i*_***	***R*^2^(adj.)**
1	Time	476.80	0	0.07
2	Ecosystem compartment	227.95	0	0.45
3	Time + Ecosystem compartment	147.60	0	0.60
4	Time × Ecosystem compartment	**0.00**	**1**	**0.86**

*The minimum-adequate model is highlighted in bold. ΔAIC is the difference in AIC (Akaike Information Criterion) with respect to the best-fitting model (lowest value of AIC). The Akaike weight (w_i_) is the relative likelihood of each model being the best-fitting model, given the complete set of candidate models. R^2^ is the variance explained by the model*.

**Table 3:** Minimum-adequate generalised mixed-effects model (GAMM) for the effects of ecosystem compartment and time on ^15^N enrichment.

	**Estimate**	**SE**	**t/*F*-value**	**edf**	***P*-value**
**Parametric coefficients:**					
Intercept	3.941	0.09	41.84		<0.001
Compartment (moss layer)	1.358	0.08	17.80		<0.001
Compartment (O-horizon)	−0.349	0.08	−4.47		<0.001
Compartment (A-horizon)	−2.321	0.08	−29.76		<0.001
Compartment (B-horizon)	−3.364	0.08	−43.41		<0.001
**Smooth terms:**					<0.001
*f* (Time) ^*^ Compartment (above-ground biomass)			20.61	3.64	<0.001
*f* (Time) ^*^ Compartment (moss layer)			214.69	1.00	<0.001
*f* (Time) ^*^ Compartment (O-horizon)			12.41	1.90	<0.001
*f* (Time) ^*^ Compartment (A-horizon)			4.03	3.02	0.006
*f* (Time) ^*^ Compartment (B-horizon)			16.36	2.97	<0.001
**Variance components:**					
SD (plot)	0.226				
SD (residuals)	0.439				
φ (residuals)	0.197				

*The intercept corresponds to the above-ground biomass compartment. The estimated degrees of freedom (edf) indicate the amount of smoothing. SE, Standard error (of the mean); SD, Standard deviation; ϕ, Estimated temporal correlation of the residuals (AR-1)*.

### ^15^N tracer recovery

**Figure 2:**
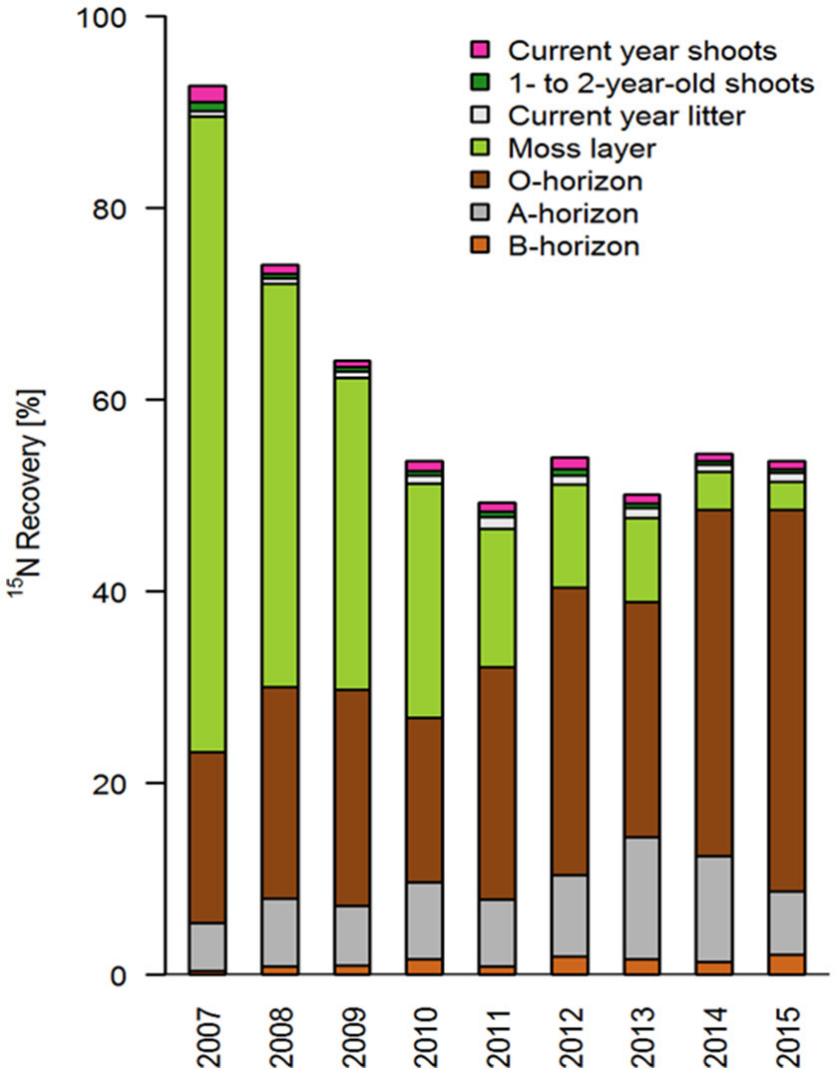
Temporal variation (2007–2015) of relative ^15^N recovery (% of the total ^15^N tracer applied) in different ecosystem compartments (*n* = 7 per compartment and year).

Total ^15^N recovery was highest in the first year (about 93%); it then (almost linearly) decreased until 2010 to a value of about 54% (Figure 2; Table 4). Interestingly, recovery rates remained largely constant in subsequent years and achieved values between 49 and 54% until 2015. The recovery rates calculated increase by about 7% if the compartments which were not sampled annually are taken into account: “*Calluna* biomass older than 2 years” (about 2.5%), and “*Calluna* roots” (about 4.2%; means across sampling years; Table 4).

**Table 4:** ^15^N enrichment [‰] and ^15^N recovery [% of the total ^15^N applied] of the sampled ecosystem compartments (means, 1 SE in brackets) during the study period 2007–2015.

**Year**	**Current year shoots**	**1- to 2-year-old shoots**	***Calluna* shoots/woody parts older than 2 years**	***Calluna* roots**	**Current year litter**	**Moss layer**	**O-horizon**	**A-horizon**	**B-horizon**	**total**
**^15^N ENRICHMENT [%]**										
2007	135.72	117.00			61.27	746.60	24.38	2.81	0.46	
	(25.42)	(23.10)			(25.18)	(106.95)	(4.03)	(0.34)	(0.22)	
2008	72.03	62.69			43.21	544.87	33.55	5.15	1.74	
	(10.72)	(11.54)			(10.10)	(56.34)	(5.98)	(1.00)	(0.64)	
2009	54.44	49.63			48.18	442.26	35.61	3.99	1.66	
	(5.91)	(6.92)			(11.67)	(64.83)	(5.78)	(0.50)	(0.62)	
2010	62.54	54.18			46.51	346.97	22.59	4.72	2.19	
	(8.17)	(6.62)			(9.01)	(33.39)	(3.58)	(1.11)	(0.63)	
2011	57.85	55.23			44.63	185.41	29.86	4.55	1.57	
	(5.64)	(5.48)			(7.62)	(19.09)	(2.47)	(1.01)	(0.43)	
2012	73.48	67.00			38.03	149.50	42.43	6.39	2.64	
	(9.11)	(7.97)			(6.85)	(13.27)	(7.18)	(1.05)	(0.68)	
2013	56.05	48.56			36.45	117.21	33.12	6.98	2.02	
	(6.37)	(5.36)			(6.44)	(10.07)	(3.44)	(0.44)	(0.58)	
2014	42.47	38.91	45.77	66.47	31.09	57.55	50.61	7.24	1.89	
	(5.88)	(4.97)	(5.16)	(10.69)	(5.76)	(7.35)	(7.69)	(1.10)	(0.43)	
2015	40.26	39.47	41.81	67.49	27.18	46.97	63.35	5.19	3.34	
	(6.61)	(5.64)	(6.21)	(5.73)		(7.11)	(6.40)	(1.07)	(0.78)	
**^15^N RECOVERY [%]**										
2007	1.69	0.89			0.56	66.38	17.80	5.01	0.43	92.75
	(0.41)	(0.21)			(0.21)	(10.30)	(2.98)	(1.36)	(0.24)	
2008	0.96	0.44			0.62	42.08	22.08	7.05	0.89	74.13
	(0.14)	(0.08)			(0.14)	(5.92)	(3.41)	(1.46)	(0.18)	
2009	0.74	0.40			0.74	32.48	22.55	6.28	0.96	64.15
	(0.08)	(0.05)			(0.16)	(5.42)	(3.10)	(1.66)	(0.29)	
2010	1.04	0.48			0.88	24.44	17.11	8.08	1.62	53.65
	(0.14)	(0.07)			(0.17)	(2.73)	(2.74)	(2.68)	(0.59)	
2011	0.94	0.54			1.25	14.42	24.27	6.91	0.94	49.26
	(0.10)	(0.06)			(0.22)	(1.37)	(2.59)	(1.93)	(0.19)	
2012	1.24	0.62			0.94	10.76	30.05	8.43	1.95	53.99
	(0.14)	(0.09)			(0.16)	(1.35)	(3.63)	(1.94)	(0.48)	
2013	0.91	0.42			1.04	8.85	24.52	12.73	1.63	50.11
	(0.10)	(0.06)			(0.20)	(0.81)	(3.14)	(2.55)	(0.37)	
2014	0.74	0.33	2.49	3.60	0.80	3.97	36.11	11.02	1.39	54.37
	(0.10)	(0.05)	(0.25)	(0.57)	(0.14)	(0.52)	(6.02)	(2.99)	(0.58)	60.47^*^
2015	0.83	0.41	2.59	4.86	0.93	2.91	39.89	6.61	2.08	53.65
	(0.13)	(0.07)	(0.54)	(0.77)	(0.15)	(0.46)	(4.89)	(1.46)	(0.55)	61.10^*^

* *Total recovery of all compartments, including compartments not sampled annually (Calluna older than 2 years and roots)*.

**Figure 3:**
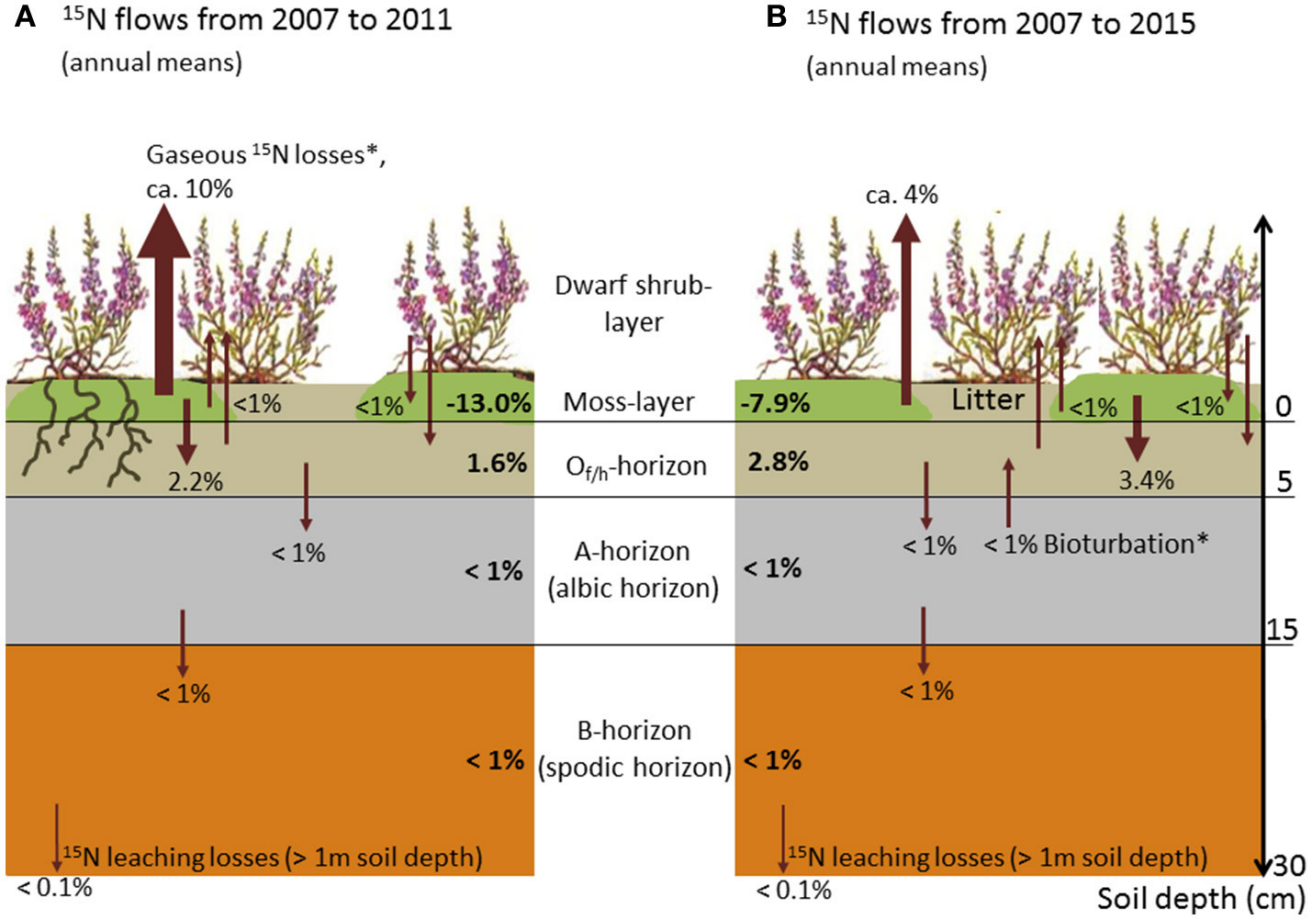
Schematic illustration of annual mean flows of ^15^N tracer between ecosystem compartments (arrows) and related annual shifts in ^15^N recovery (bold numbers; positive and negative values indicate annual gains and losses, respectively; all numbers in % of the total amount of tracer applied). **(A)** Annual mean flows for the time span 2007–2011, and **(B)** annual mean flows for the time span 2007–2015. The thickness of arrows roughly indicates the mass of between-compartment transported ^15^N tracer (^*^ = hypothesized flows based on quantified ^15^N mass balances).

The moss layer proved to be the most important short-term sink and longer-term source for applied ^15^N, and showed a distinct decrease in recovery rates within the first 5 years (from 66% in 2007 to 14% in 2011; Figures 2, 3). This loss of ^15^N from the moss layer was not compensated for by a corresponding increase in recovery rates in any other compartment (Figure 3). We did find increasing recovery rates in the O- and A-horizons in the course of the experiment (e.g., from 18 to 40% for the O-horizon). This increase corresponded to about 50% of the total ^15^N losses from the moss layer between 2007 and 2015 (Figure 2). For the B-horizon, we found a slight but continuous increase in ^15^N recovery, with a maximum of 2.1% in the last year of the experiment.

Recovery rates in both the current year and 1- to 2-year-old shoots of *Calluna* were negligibly low in all study years, with values below 2%, and in most years even below 1% (Figures 2, 3, Table 4). Accordingly, we also found low recovery rates for the annual litter of *Calluna*, which amounted to ≤1%. In addition, leaching losses of ^15^N proved to be very low, as indicated by an annual recovery of 0.03% of the total ^15^N applied. Thus, leaching losses were even lower than at the beginning of the experiment (0.05% in 2007).

## Discussion

### Decadal-scale allocation patterns of N

Our findings support our first hypothesis that the moss layer acts as both an important short-term sink and a longer-term source of captured ^15^N tracer. The moss layer thus represents a crucial link between airborne N inputs and the allocation of N to the heathland's N cycle (in term of its capture-release function). The moss layer's ability to store incoming N in the short term may be related to the fact that mosses represent ectohydric plants that absorb dissolved nutrients at the cell surface (Proctor, 2008). The decadal-scale availability and release of N could be attributable to a longer-term sequestration of N due to N assimilation by mosses and the microbial community associated with the moss phytomass (Gordon et al., 2001; Tye et al., 2005).

An important finding in terms of the N balance of the ecosystem was that, during the first 4 years of the experiment, ^15^N losses from the moss layer were not compensated for by a corresponding increase in recovery rates in any other compartment. These losses accounted for more than 30% of the total ^15^N applied and suggest that N losses from the moss layer in gaseous forms are very likely (Startsev and Lieffers, 2007; for a further discussion of this aspect see paragraph ‘Leaching losses and putative flows’ below). In contrast, the largely constant recovery rates found from 2010 onwards point to a conservative N cycling with marginal ^15^N losses from the N pool of the ecosystem in the long term (Tye et al., 2005).

Our second hypothesis was not confirmed, since the O-horizon (and not the B-horizon; see discussion below) proved to be an important long-term sink for sequestered ^15^N. In this horizon, recovery rates more than doubled in the course of the experiment. Several mechanisms may explain the N-flow to this compartment: Firstly, a passive downward transport of N from the moss to the organic layer via leaching might have taken place (Startsev et al., 2008), particularly at the beginning of the experiment. Secondly, decomposition processes of the lower and dead segments of the bryophytes, which sequestered ^15^N in biomass after tracer addition in 2007, are likely to have contributed to an increase in ^15^N recovery in the O-horizon over time (Aldous, 2002). Thirdly, processes such as mineralisation of *Calluna* litter and leaking bryophyte cells (as a result of desiccation-rehydration processes) could have contributed to increasing ^15^N recovery rates in the O-horizon (Bates, 2008; Friedrich et al., 2011b). Downward allocation processes of N (both as particular and dissolved organic matter) in the soil profile also explain an increasing recovery of ^15^N in the A-horizon. These findings coincide with observations in grasslands and forests, which showed the upper soil horizons (and the O-horizons in particular) to be important long-term sinks for airborne N loads (Gerzabek et al., 2004; Nadelhoffer et al., 2004; Templer et al., 2012; Gurmesa et al., 2016). High N retention in the O-horizon was also reported from heathland studies (although not based on tracer analyses; Kristensen and McCarty, 1999; Kristensen, 2001; Schmidt et al., 2004; Pilkington et al., 2005), and mainly attributed to an immobilisation of N by the microbial biomass present in these horizons (Jonasson et al., 1996; Nordin et al., 2004). A reverse trend in N recovery in the O- and A-horizons observed in the last 2 years of the experiment suggests that upward transports of N in the soil profile are also possible, probably due to bioturbation via root growth or soil invertebrates (Gabet et al., 2003).

Despite a continuous increase in ^15^N enrichment and recovery in the podzol's B-horizon, we found no clear support for our assumption that considerable quantities of N were sequestered in this horizon. We consider a decadal-scale recovery of about 2% too low to cause an ecologically relevant N withdrawal from the N cycle (which would be related to an N storage in insoluble and not readily bio-available forms; Hagedorn et al., 2005). Since the formation of a podzol spodic horizon is assumed to take centuries (Brady and Weil, 2008), an accumulation of N up to one ton per ha in this horizon (as found for our study area; Friedrich et al., 2011b) might require timespans far beyond the observation time of the present study. This assumption would be backed by the observed ^15^N accumulation rates, given that these could be extrapolated to a centennial scale.

Recovery of ^15^N in above- and below-ground biomass of *Calluna* was very low compared to recovery rates in the moss and organic layer. We hypothesise that soil microbes in the organic layer were the superior competitors for N (Andresen et al., 2008), and only small amounts of ^15^N remained available for plant uptake (Schimel and Bennett, 2004). This was demonstrated by Larsen et al. (2012) in a subarctic heathland, where 65% of the applied ^15^N was sequestered by soil microbes. In this context, it is important to note that high immobilisation rates by soil microbes are also indicative of a low N saturation level (Curtis et al., 2005).

### Leaching losses and putative flows

^15^N losses via leaching proved to be negligibly low. This is an important indication of conservative N cycles (Tye et al., 2005) and contradicts the notion that the heathlands of the study area have achieved an advanced stage of N saturation (Aber et al., 1998), despite decades of N inputs beyond critical load thresholds. Obviously, the sites analysed still have a high capacity to sequester pollutant N (Choudhary et al., 2016), suggesting that a constant proportion of incoming N enters the heathland's N cycle and contributes to a slow but steady increase in the N level (De Schrijver et al., 2011). This confirms our third hypothesis.

In contrast to the observed accumulation of N, the first 4 years of the experiment (2007–2010) also exhibited a considerable loss of ^15^N. Tracer mass balances suggest that this loss was attributable to a leak of N from the moss layer in gaseous forms (Startsev and Lieffers, 2007). High N losses from bryophyte layers due to denitrification have been found for different species and ecosystems, and increase with increasing humidity (e.g., with water saturation of the moss layer after precipitation events), temperature, and N deposition (Laverman et al., 2000; Opelt and Berg, 2004; Lenhart et al., 2015). As a consequence, gaseous N losses from moss carpets are considered to have significant impacts on ecosystem N budgets (Fang et al., 2015), and also contribute to global nitrous oxide emissions from terrestrial ecosystems (Lenhart et al., 2015). For example, Calvo-Fernández et al. (2015) found high N losses as a result of denitrification in Cantabrian Mountain heathlands, when soils were water-saturated during winter. Fang et al. (2015) concluded that the effects of denitrification losses on ecosystem N balances are often underestimated. NH_3_ volatilisation might also have contributed to ^15^N losses from the moss layer (Mahendrappa and Ogden, 1973; Startsev et al., 2008). Further mechanisms of N losses could be lateral N transport by mycorrhiza or soil fauna. However, since the natural ^15^N abundances in non-labelled subplots remained unchanged in the course of the experiment, we consider these losses (including conceivable lateral transports due to surface run-off) to be of minor importance. Low lateral transports also might be related to high absorption rates of ammonium, which is the prevailing N_inorg_ form at podzol sites with strongly acidic soil condition (cf. study site description). Due to the avoidance of destructive sampling, N contents of roots below a soil depth of 15 cm were not quantified. Since *Calluna* is considered a shallow-rooted plant (with more than 90% of root biomass being located in the humus horizons; Gimingham, 1972), and CN ratios of deep rooting woody roots are exceptionally high (>120; own unpublished data), we consider the N pool related to this compartment to be very low. We also rule out a significant effect of N deposition on ^15^N recovery patterns, since N deposition is evenly distributed across the study area, and thus between-plot differences in N deposition are low (including differences in N forms and isotope signatures; cf. deposition measurements of Niemeyer et al., 2005).

In conclusion, we found evidence that about 60% of the added ^15^N remained in the N cycle of the ecosystem at least for about one decade. In this context moss layers may play a crucial functional role for the N cycle of ecosystems: On the one hand, moss layers may contribute to high N losses by fostering denitrification processes (particularly under oceanic to sub-oceanic climates), and thus may decelerate N eutrophication processes in ecosystems. On the other hand, mosses represent a considerable short-term sink for incoming N which is released to the N cycle of an ecosystem in the long term. As a result, a considerable proportion of airborne N inputs can accumulate in different ecosystem compartments. Since the O-horizon proved to be an important long-term sink, it is likely that N captured by this horizon remains bio-available in the long term.

This has important implications for both critical load estimates and the development of management strategies to counteract airborne N inputs. A continuous accumulation of bio-available N (even under low N inputs) would implicate that critical load revisions need to account for cumulative effects of (low) N additions into ecosystems (Payne et al., 2011; Phoenix et al., 2012; Bähring et al., 2017). Mitigation strategies (in terms of management) should comprise high-intensity measures (e.g., choppering and sod-cutting; Härdtle et al., 2006; Frouz et al., 2009) as an important means to achieve balanced N budgets in the long term, because only these measures affect upper soil horizons with high contents of accumulated N.

## Author contributions

UF, WH, and GvO conceived of the study. All authors contributed to the field and lab work, the analysis and interpretation of data, and the writing of the manuscript. All authors approved the final version of the manuscript.

### Conflict of interest statement

The authors declare that the research was conducted in the absence of any commercial or financial relationships that could be construed as a potential conflict of interest.
